# Accuracy of two pulse-oximetry measurements for INTELLiVENT-ASV in mechanically ventilated patients: a prospective observational study

**DOI:** 10.1038/s41598-021-88608-7

**Published:** 2021-04-26

**Authors:** Shinshu Katayama, Jun Shima, Ken Tonai, Kansuke Koyama, Shin Nunomiya

**Affiliations:** Division of Intensive Care, Department of Anaesthesiology and Intensive Care Medicine, Jichi Medical University School of Medicine, 3311-1, Yakushiji, Shimotsuke, Tochigi 329-0498 Japan

**Keywords:** Respiration, Medical research, Signs and symptoms

## Abstract

Recently, maintaining a certain oxygen saturation measured by pulse oximetry (SpO_2_) range in mechanically ventilated patients was recommended; attaching the INTELLiVENT-ASV to ventilators might be beneficial. We evaluated the SpO_2_ measurement accuracy of a Nihon Kohden and a Masimo monitor compared to actual arterial oxygen saturation (SaO_2_). SpO_2_ was simultaneously measured by a Nihon Kohden and Masimo monitor in patients consecutively admitted to a general intensive care unit and mechanically ventilated. Bland–Altman plots were used to compare measured SpO_2_ with actual SaO_2_. One hundred mechanically ventilated patients and 1497 arterial blood gas results were reviewed. Mean SaO_2_ values, Nihon Kohden SpO_2_ measurements, and Masimo SpO_2_ measurements were 95.7%, 96.4%, and 96.9%, respectively. The Nihon Kohden SpO_2_ measurements were less biased than Masimo measurements; their precision was not significantly different. Nihon Kohden and Masimo SpO_2_ measurements were not significantly different in the “SaO_2_ < 94%” group (*P* = 0.083). In the “94% ≤ SaO_2_ < 98%” and “SaO_2_ ≥ 98%” groups, there were significant differences between the Nihon Kohden and Masimo SpO_2_ measurements (*P* < 0.0001; *P* = 0.006; respectively). Therefore, when using automatically controlling oxygenation with INTELLiVENT-ASV in mechanically ventilated patients, the Nihon Kohden SpO_2_ sensor is preferable.

*Trial registration* UMIN000027671. Registered 7 June 2017.

## Introduction

Avoiding both hypoxemia and hyperoxemia is vital in critically ill patients^1–4^. Because hypoxemia poses the risk of tissue hypoxia, adequate oxygen should be prescribed for ventilation. In contrast, hyperoxemia has been associated with increased mortality^5^ and fewer ventilator-free days^6^. Pulse oximetric oxygen saturation (SpO_2_) monitor is widely used in clinical situation to monitor hypoxemia. However, hyperoxemia is frequently under-recognized when PaO_2_ is over 100 mmHg, because SpO_2_ is difficult to determine the level of PaO_2_ in this situation. In regard to the oxygen delivery, it is recommended that arterial oxygen saturation (SaO_2_) be kept at a certain range in mechanically ventilated patients. Recently, a multicentre randomised trial suggested that maintaining the oxygen saturation measured by pulse oximetry (SpO_2_) at 97% might be ideal for managing critically ill patients^7^. From the British Thoracic Society guidelines, the target saturation range for acutely ill patients who are not at risk of hypercapnic respiratory failure is 94–98%^8^. Despite the control and maintenance of adequate oxygen saturation targets, hyperoxemia has been reported in some critically ill patients^9^; approximately one out of three patients were managed with SpO_2_ > 97%. One reason for unresolved hyperoxemia was the fluctuations in the oxygenation status. In mechanically ventilated patients, despite setting an adequate SpO_2_ range, SpO_2_ can easily change due to posture, respiratory pattern, and the presence of airway secretions. Thus, it might be difficult to strictly control the oxygenation status within the ideal SpO_2_ range manually.

INTELLiVENT-ASV is one of the unique ventilator modes which available only for Hamilton Medical ventilators. It has the original function which allow to monitor patients’ SpO_2_ and end-tidal carbon dioxide (E_T_CO_2_) and automatically control the delivered fraction of inspired oxygen (F_I_O_2_) and minute ventilation volume^10,11^. The automatic F_I_O_2_ control function can be used with the SpO_2_ monitoring probe attached to the ventilator. Generally, SpO_2_ is controlled from 93 to 97% in normal lung setting by adjusting F_I_O_2_ between 0.21 and 1.0, but SpO_2_ target range can also easily alter depend on each patients’ clinical situation. Because INTELLiVENT-ASV is able to monitor and adjust oxygen breath by breath continuously, it is easier to use INTELLiVENT-ASV than a physician-driven control to maintain an appropriate SpO_2_ range. However, it is unclear how accurate the SpO_2_ measured during INTELLiVENT-ASV compared to the actual SaO_2_. Several studies have suggested that SpO_2_ measurements tend to overestimate the oxygenation status in critically ill patients^12–14^. In addition, few studies have evaluated the relationship between actual SpO_2_ and SaO_2_ using either the Nihon Kohden or the Masimo pulse oximeter, which are the only two available for INTELLiVENT-ASV^15^.

In this study, we aimed to compare the accuracy of bias and precision of the two pulse oximeters in mechanically ventilated patients in an intensive care unit (ICU) setting. In addition, we evaluated the accuracy of SpO_2_ measurements in various SaO_2_ range categories.

## Results

### Enrolment and baseline characteristics

**Figure 1:**
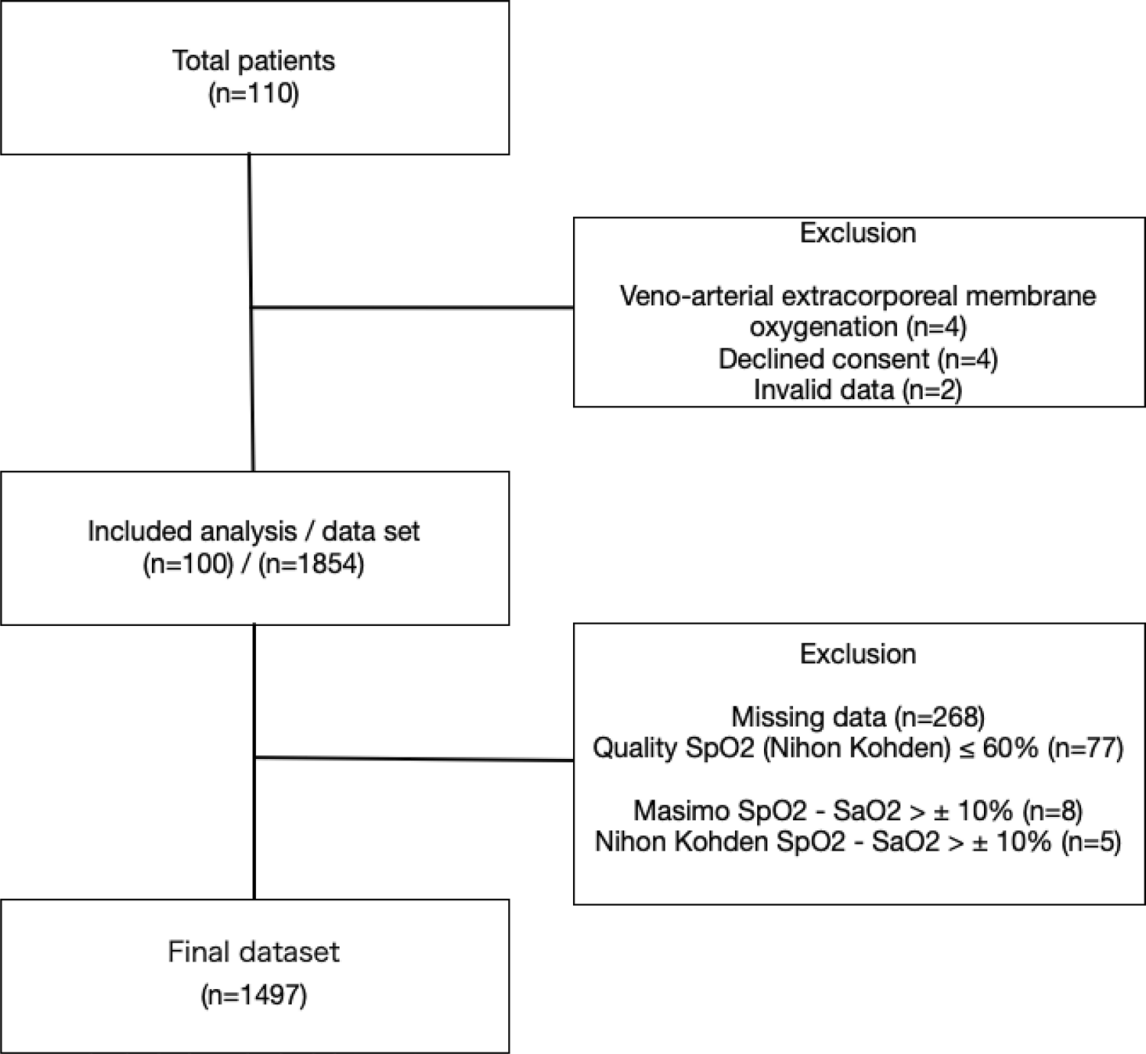
Patients’ flow chart.

In total, the results of 1854 blood gas analyses performed in 100 patients ventilated with HAMILTON G5 ventilator (Hamilton Medical AG, Rhäzüns, Switzerland) were considered for evaluation (Fig. 1). Of these results, the following were excluded: those with missing data on the Nihon Kohden and Masimo SpO_2_ measurements (n = 268), those in which the Nihon Kohden SpO_2_ measurements were ≤ 60% (n = 77), those in which the difference in value between the Nihon Kohden SpO_2_ measurement and the actual SaO_2_ was > 10% (n = 5), and those in which the difference in value between the Masimo SpO_2_ measurement and the actual SaO_2_ was > 10% (n = 8).

Table 1 shows the characteristics of patients ventilated with a HAMILTON G5 ventilator. The mean value of the blood gas analyses was 15.0 ± 15.4, mean Acute Physiology and Chronic Health Evaluation II score was 24.6 ± 8.3, and the mean duration of mechanical ventilation was 9.0 ± 7.4 days. The arterial oxygen tension (PaO_2_)/F_I_O_2_ ratio was 249.6, categorised into PaO_2_/F_I_O_2_ > 300 (26%), 200 < PaO_2_/F_I_O_2_ ≤ 300 (36%), 100 < PaO_2_/F_I_O_2_ ≤ 200 (22%), and PaO_2_/F_I_O_2_ ≤ 100 (16%). ICU and hospital mortality rates were 5.0% and 16.0%, respectively.

**Table 1:** Patient characteristics.

Mean ± SD	n = 100
Age (years)	63.3 ± 15.3
Height (cm)	158.7 ± 9.3
Weight (kg)	60.1 ± 15.9
Body mass index	23.7 ± 5.1
Male	48 (48%)
Number of Blood gas analysis (25th–75th percentile)	15.0 ± 15.4
Acute Physiology and Chronic Health Evaluation II	24.6 ± 8.3
**Classification of disease**	
Respiratory	36 (36%)
Cardiovascular	9 (9%)
Gastrointestinal	35 (35%)
Neurological	9 (9%)
Haematological	2 (2%)
Trauma	3 (3%)
Gynaecology	2 (2%)
Other	4 (4%)
**Co-morbidity**	
Hypertension	43 (43%)
Ischaemic heart disease	8 (8%)
Chronic heart failure	6 (6%)
Arrhythmia	8 (8%)
COPD	7 (7%)
Cerebrovascular accident	6 (6%)
Diabetes mellitus	20 (20%)
Chronic kidney disease on haemodialysis	5 (5%)
hepatic disease	11 (11%)
**Blood gas analysis (including all analyses, n = 1497)**	
pH	7.426 ± 0.081
PaO_2_, mmHg	92.5 ± 35.1
PaCO_2_, mmHg	40.4 ± 8.9
Haemoglobin, g/dL	9.3 ± 1.8
SaO_2_, %	95.7 ± 2.9
MV duration, days	9.0 ± 7.4
PaO_2_/F_I_O_2_ (day 1)	249.6 ± 143.6
PaO_2_/F_I_O_2_ > 300	26 (26%)
200 < PaO_2_/F_I_O_2_ ≤ 300	36 (36%)
100 < PaO_2_/F_I_O_2_ ≤ 200	22 (22%)
PaO_2_/F_I_O_2_ ≤ 100	16 (16%)
Veno-venous extracorporeal membrane oxygenation	2 (2%)
ICU days	10.3 ± 7.6
ICU mortality	5 (5%)
Hospital mortality	16 (16%)

*COPD* chronic obstructive pulmonary disease, *ICU* intensive care unit, *SaO*_*2*_ arterial oxygen saturation, *SpO*_*2*_ pulse-oximetric oxygen saturation, *PaCO*_*2*_ arterial carbon dioxide tension, *PaO*_*2*_ arterial oxygen tension.

### Bland–Altman plot comparing SpO_2_ and SaO_2_

**Figure 2:**
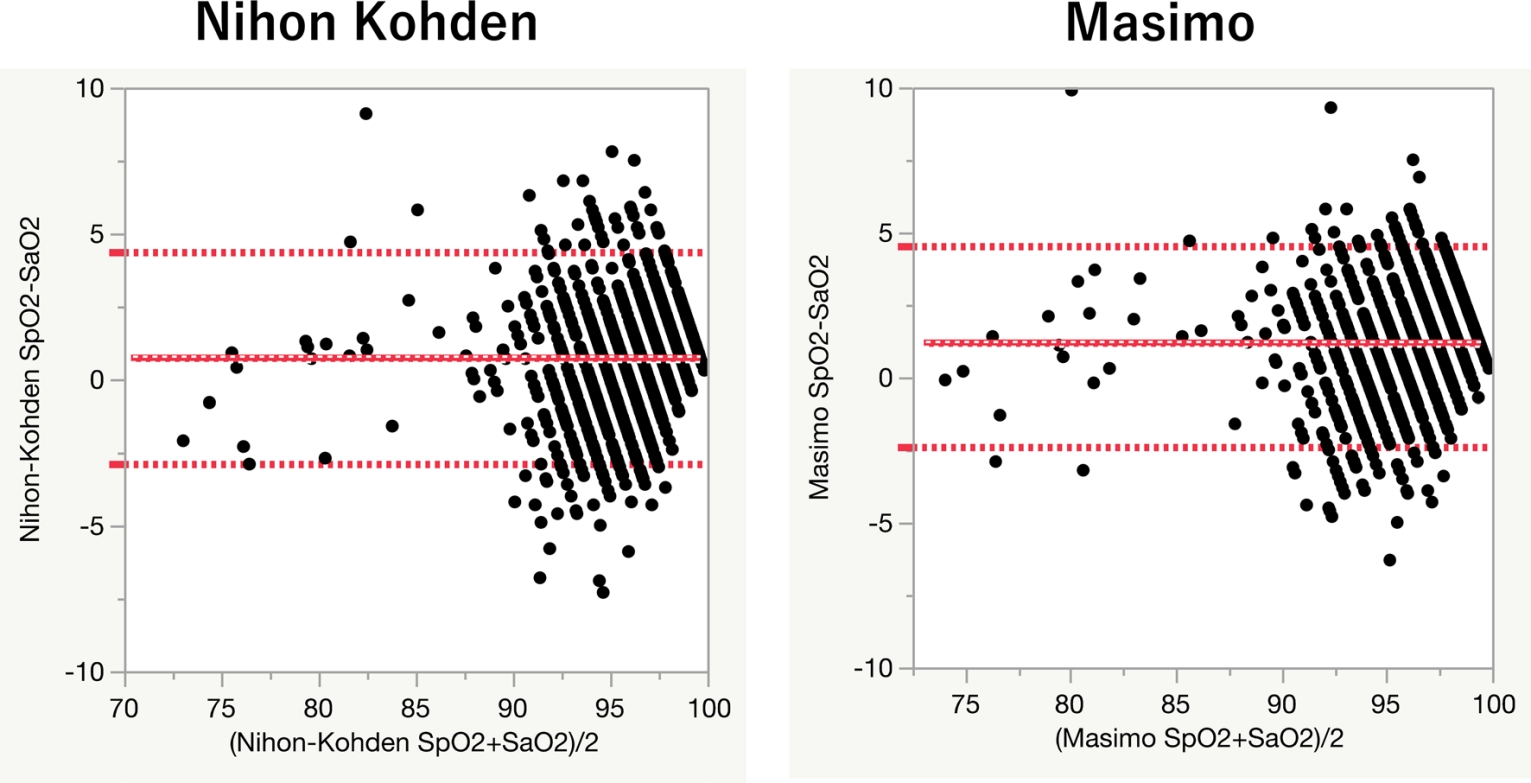
Bland–Altman plot comparing SaO_2_ and SpO_2_. The horizontal axis represents (SpO_2_ + SaO_2_)/2. The vertical axis represents SpO_2_ – SaO_2_. The middle horizontal red line represents bias. The upper horizontal red dotted line represents the upper limits of agreement, and the lower horizontal red dotted line represents the lower limits of agreement. Black dots represent each test result. The left figure portrays Nihon Kohden SpO_2_, and the right figure portrays Masimo SpO_2_.

A Bland–Altman plot was generated to evaluate bias and precision (Fig. 2). Mean SpO_2_ was 96.4% using the Nihon Kohden and 96.9% using the Masimo monitor. The bias was lower in the Nihon Kohden SpO_2_ measurements than in the Masimo measurements, although the precision was not significantly different (mean ± SD, 0.72 ± 1.86% vs 1.17 ± 1.68%, respectively: Table 2).

**Table 2:** Bias and precision for each pulse oximeter (n = 10 000).

	Mean (95% CI)
**Blood gas analysis**	
SaO_2_	95.72% (95.57–95.86)
**Nihon Kohden SpO**_**2**_	
SpO_2_	96.4% (96.3–96.6)
Bias (SpO_2_—SaO_2_)	0.72% (0.62–0.81)
Precision	1.86 (1.78–1.95)
Upper limits of agreement	4.37 (4.11–4.63)
Lower limits of agreement	−2.93 (−3.20 to −2.69)
**Masimo SpO**_**2**_	
SpO_2_	96.9% (96.7–97.1)
Bias (SpO_2_–SaO_2_)	1.17% (1.08–1.26)
Precision	1.68 (1.61–1.77)
Upper limits of agreement	4.46 (4.24–4.73)
Lower limits of agreement	−2.12 (−1.90 to −2.39)

*CI* confidential interval, *SaO*_*2*_ arterial oxygen saturation, *SpO*_*2*_ pulse-oximetric oxygen saturation.

We also analysed all raw data, including outliers. The bias improved slightly in the Masimo; however, the precision deteriorated in the Masimo measurements compared to the Nihon Kohden measurements (0.72 ± 2.07% vs 1.08 ± 2.42%) (see Supplementary Table S1 and Supplementary Fig. S1).

### Differences between SaO_2_ and pulse oximeters’ SpO_2_ among SaO_2_ categories

**Figure 3:**
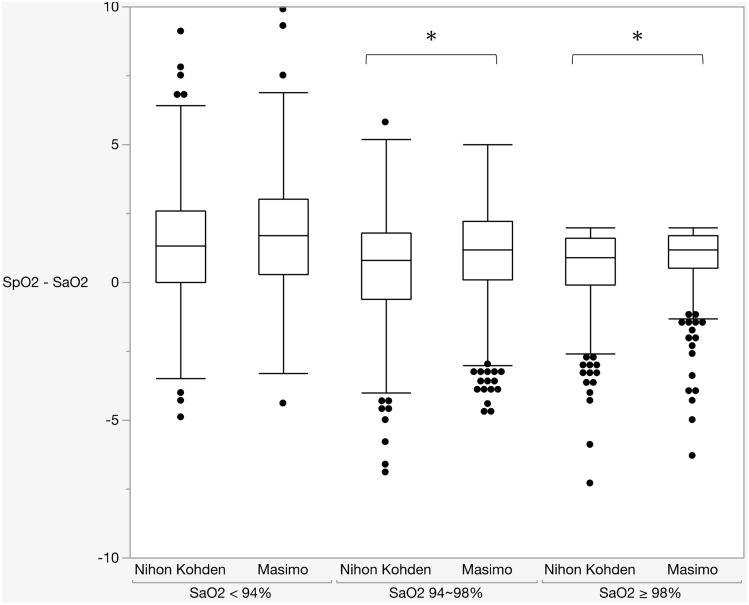
Differences in the pulse oximeters’ SpO_2_ measurements among SaO_2_ categories. Three SaO_2_ categories were established (SaO_2_ < 94%, 94% ≤ SaO_2_ < 98%, SaO_2_ ≥ 98%). The vertical axis represents SpO_2_ – SaO_2_, the horizontal axis represents Nihon Kohden SpO_2_ and Masimo SpO_2_ for each of the three groups. There were significant differences in the groups with SaO_2_ ≥ 94%. However, there was no significant difference in the SaO_2_ < 94% group. *: *P* < 0.005.

Among the three SaO_2_ range categories (SaO_2_ < 94%, 94% ≤ SaO_2_ < 98%, and SaO_2_ ≥ 98%), we evaluated the differences in SpO_2_ measurements by both monitors within each group (Fig. 3 and Table 3). In the “SaO_2_ < 94%” group, there was no significant difference between the Nihon Kohden SpO_2_ measurements and the Masimo measurements (1.41 ± 2.28% vs 1.74 ± 2.15%, *P* = 0.083). However, in the “94% ≤ SaO_2_ < 98%” and “SaO_2_ ≥ 98%” groups, there were significant differences between the Nihon Kohden SpO_2_ measurements and the Masimo measurements (0.57 ± 1.77% vs 1.10 ± 1.60%, *P* < 0.0001; 0.51 ± 1.49% vs 0.84 ± 1.28%, *P* = 0.006; respectively), although there was a slight difference of -3% or more compared to actual SaO_2_ and the difference was larger than Masimo SpO_2_ in “SaO_2_ ≥ 98%” group (Table 3).

**Table 3:** Differences in SpO_2_ measurements between pulse oximeters.

	Mean ± SD (95% CI)	*P*-value
**SaO**_**2**_ < 94% (Blood gas analysis = 274)		
Nihon Kohden	1.41 ± 2.28% (1.14–1.68)	0.083
Masimo	1.74 ± 2.15% (1.49–2.00)	
**94% ≤ SaO**_**2**_ < 98% (Blood gas analysis = 952)		
Nihon Kohden	0.57 ± 1.77% (0.46–0.69)	< 0.0001
Masimo	1.10 ± 1.60% (1.00–1.20)	
**SaO**_2_ ≥ 98% (Blood gas analysis = 271)		
Nihon Kohden	0.51 ± 1.49% (0.33–0.69)	0.006
Masimo	0.84 ± 1.28% (0.68–0.99)	

*CI* confidence interval, *SaO*_*2*_ arterial oxygen saturation, *SD* standard deviation, *SpO*_*2*_ pulse-oximetric oxygen saturation.

### Area under the receiver operating characteristic curve to detect SaO_2_ < 90% and SaO_2_ ≥ 98%

**Figure 4:**
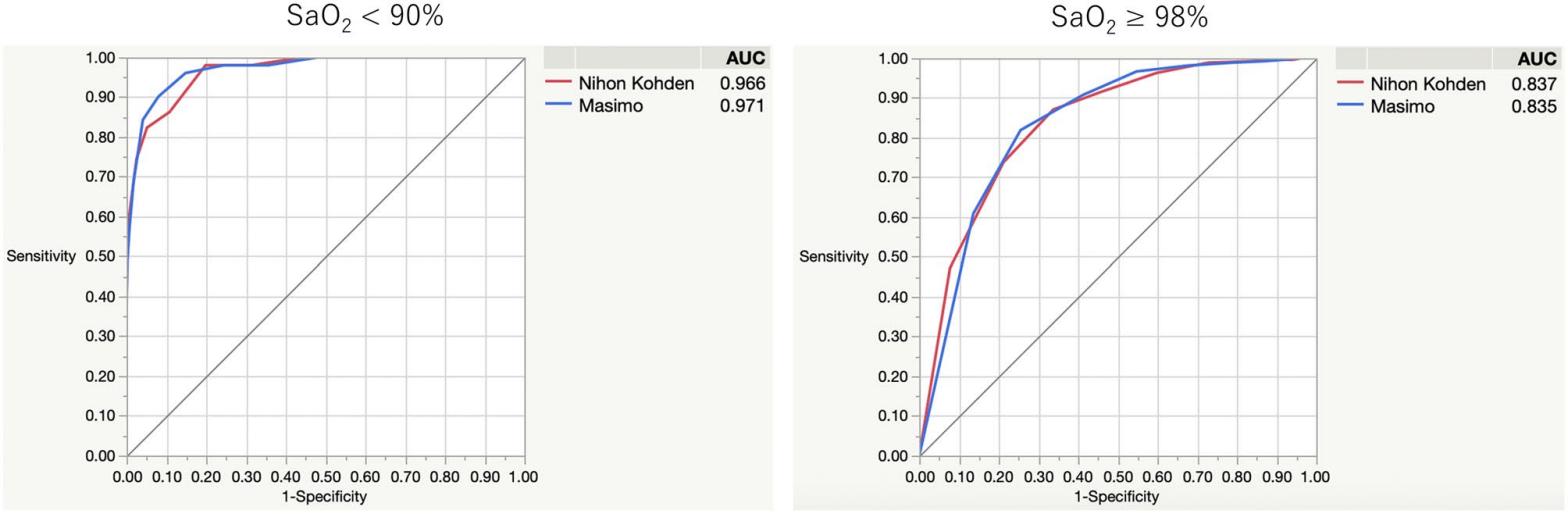
AUC comparison between Nihon Kohden SpO_2_ and Masimo SpO_2_ for patients with SaO_2_ < 90% and SaO_2_ ≥ 98%. The blue line represents Masimo SpO_2_ measurements, and the red line represents Nihon Kohden SpO_2_. The vertical axis represents true positive rate calculated by sensitivity, and the horizontal axis represents false positive rate calculated by (1—specificity). To detect SaO_2_ < 90%, the area under the receiver operating characteristic curves (AUCs) were 0.966 using the Nihon Kohden and 0.971 using the Masimo monitor. When cut off point was set at SpO_2_ 90% in Nihon Kohden and Masimo, its sensitivity and specificity were 62.8% and 99.0% in Nihon Kohden SpO_2_, and 56.9% and 99.3% in Masimo SpO_2_, respectively. There was no statistically significant difference in the AUCs between both monitors (*P* = 0.530). Regarding SaO_2_ ≥ 98%, the AUCs were 0.837 using the Nihon Kohden and 0.835 using the Masimo monitor. When cut off point was set at SpO_2_ 98% in Nihon Kohden and Masimo, its sensitivity and specificity were 87.1% and 66.2% in Nihon Kohden SpO_2_, and 90.8% and 58.7% in Masimo SpO_2_, respectively. However, there was no statistically significant difference in the AUCs of both monitors (*P* = 0841). AUC, receiver operating characteristic curve.

We evaluated each pulse oximeter’s ability to detect SaO_2_ < 90%. The area under the receiver operating characteristic curves (AUCs) were 0.966 using the Nihon Kohden and 0.971 using the Masimo monitor. When cut off point was set at SpO_2_ 90% in Nihon Kohden and Masimo, its sensitivity and specificity were 62.8% and 99.0% in Nihon Kohden SpO_2_, and 56.9% and 99.3% in Masimo SpO_2_, respectively. There was no statistically significant difference in the AUCs between both monitors (*P* = 0.530). Regarding SaO_2_ ≥ 98%, the AUCs were 0.837 using the Nihon Kohden and 0.835 using the Masimo monitor. When cut off point was set at SpO_2_ 98% in Nihon Kohden and Masimo, its sensitivity and specificity were 87.1% and 66.2% in Nihon Kohden SpO_2_, and 90.8% and 58.7% in Masimo SpO_2_, respectively. However, there was no statistically significant difference in the AUCs of both monitors (*P* = 0.841; Fig. 4).

## Discussion

In this study, we found that Nihon Kohden SpO_2_ measurements presented a lower bias than did Masimo SpO_2_ measurements compared with the actual SaO_2_. The precision and correlation coefficients of SpO_2_ measurements were similar in both devices; specifically, these trends were seen in the “SaO_2_ ≥ 94%” group. In the low SpO_2_ group, there were no significant differences between SpO_2_ values measured by both devices.

Pulse oximeters indicate the calculated oxygen saturation values using the transmitted light signals of an LED irradiated on body. The theoretical formula for determining SpO_2_ from transmitted light signals has not been established, and each manufacturer uses its own formula. Therefore, the accuracy of SpO_2_ includes two factors: the validity of the formula and deviation from the formula. Also, pulse oximeters should detect the pulsation of arterial blood, but when the other pulsatile noises such as body movement are detected, it might be cause erroneous calculation. To reduce these noises, each manufacturer uses its own original technology. The results of this study might be influenced by these technological differences between Nihon Kohden and Masimo SpO_2_.

To safely perform automatic control of a closed-loop ventilation system, it is essential to collect high-quality data continuously. When using the current INTELLiVENT-ASV, two types of SpO_2_ measuring devices are now available: the Masimo and the Nihon Kohden. A previous study reported that Masimo SpO_2_ measurements tended to overestimate SaO_2_ compared to Nihon Kohden measurements^15^. In this study, although the precisions of both devices were similar, Masimo SpO_2_ measurements were higher than Nihon Kohden SpO_2_ measurements. Specifically, Masimo SpO_2_ measurements were significantly different for SaO_2_ readings > 94%. These results suggest we should be more cautious regarding overestimation of SaO_2_ when using the Masimo SpO_2_ readings. In this regard, using the Nihon Kohden SpO_2_ monitor may be preferable with INTELLiVENT-ASV. On the other hand, the SpO_2_ measured by Nihon Kohden often showed a difference of -3% or more compared to actual SaO_2_ and the difference was larger than Masimo. It should be interpreted cautiously that the likelihood of misdetermination of hyperoxemia as normal was higher than that of Masimo.

At the low SpO_2_ range (SpO_2_ < 94%), the difference between devices was not statistically significant; concerning hypoxaemia detection, each pulse oximeter presented relatively high AUC values without a significant difference. According to a previous study, there was no significant difference between Nihon Kohden and Masimo SpO_2_ measurements in the range of 85% < SpO_2_ ≤ 90%^15^. Our result is similar; although, the relatively small number of analyses in our study might have influenced this result. In this study, both devices presented high SpO_2_ compared with actual SaO_2_. In clinical settings, it is important to check the actual SaO_2_ when SpO_2_ presented higher than expected. Also, there was a difference that Masimo's equipment had true positive rate of 90% or more when false positive rate is 10%, but Nihon Kohden has a false positive rate of 15% to raise true positive rate to 90%. The small number of this group (n = 51) might partly be affected on this result. Because we usually control SpO_2_ over 90% in critically ill patients, the number of blood gas analyses was relatively small compared with another group. Further examination is needed to evaluate this results in critically ill patients.

It is important to mount SpO_2_ sensors correctly, especially in an ICU setting. A previous study evaluated four types of pulse oximeters that could accurately detect low perfusion during motion^16^. Motion impaired the performance of all four oximeters at all ranges, with lesser accuracies observed at the lower SaO_2_ range. In contrast, at a lower perfusion, only the Nihon Kohden SpO_2_ measurements remained accurate. In addition, when the probe was not symmetrically placed, SpO_2_ measurements were inaccurate without an abnormal SpO_2_ waveform; this phenomenon is called the “penumbra effect”^17^. To avoid this problem, we selected a seal-type sensor for all patients in this study.

This study has several limitations. First, it was a single-center observational study. All patients evaluated were Asian, except for one American. Because the accuracy of a pulse oximeter is affected by race^18^, further studies are warranted. Second, the mean SaO_2_ values were in the optimal range (95.7 ± 2.9%); SaO_2_ values were distributed at higher levels with respect to hypoxaemia. Thus, it might be difficult to evaluate the accuracy of these monitors in the low SaO_2_ range group. Furthermore, we did not measure the quality and perfusion indexes of the Massimo SpO_2_ measurements, which might have affected these results. However, we recorded these data when the SpO_2_ value was stable. Third, although we defined the outlier as a difference of ≥ 10% in SpO_2_ and SaO_2_ values, its validation was unclear. However, we re-analysed the set of raw data, including these outliers, and found that the results were not affected by the exclusion of outliers. Fourth, we did not evaluate the Masimo SpO_2_ measurements without mounting it on the G5. In this study, it was difficult to mount both SpO_2_ sensors on the G5 simultaneously. However, the mechanics of each SpO_2_ sensor were almost the same, with or without mounting on the G5. Finally, we could not evaluate continuously whether SpO_2_ sensors were mounted correctly. Specifically, we did not consider the perfusion index of the Masimo. Also, we did not consider the presence of covariates, such as age, gender, and/or diseases. Further studies are needed to evaluate accuracy based on probe attachment.

Despite these limitations, this study also has several strengths. First, this is the first prospective observational study evaluating the bias and precision of pulse oximeters in critically ill mechanical ventilated patients with Hamilton ventilator. In addition, we evaluated the oxygenation status with respect to SpO_2_ ≥ 98% and SpO_2_ < 90%. To keep SaO_2_ strictly in the recommended range, it might be better to use Nihon Kohden SpO_2_ measurements due to the smaller bias compared to the Masimo SpO_2_ measurements.

## Conclusions

We found that Nihon Kohden SpO_2_ measurements presented lower bias than Masimo SpO_2_ measurements compared with the actual SaO_2_. The precision of SpO_2_ measurements was relatively similar with both devices. This study suggests that when using INTELLiVENT-ASV and selecting automatic control of oxygenation in mechanically ventilated patients, it is preferable to use Nihon Kohden SpO_2_ monitoring.

## Methods

### Study design and setting

This was a single-center, prospective, observational study conducted in the general ICU of a university hospital (Tochigi, Japan) from June 2017 to November 2018 (UMIN000027671). Patients who were ventilated with a G5 ventilator (Hamilton Medical AG, Switzerland) in the ICU were included in this study. Clinical decisions, including changing ventilation mode, were made at the discretion of the attending ICU physicians. The study protocol was approved by the Institutional Research Ethics Committee of Jichi Medical University Hospital (A18-110). Written informed consent was obtained from each participant or nearest relative when the patient was incapacitated or unconscious. All methods were performed in accordance with the relevant guidelines and regulations.

### Participants

Patients were eligible for enrolment if they were ≥ 20 years old and ventilated with a G5 during their ICU stay. The exclusion criteria were age < 20 years, quality index of Nihon Kohden SpO_2_ ≤ 60%, an unstable value of SpO_2_ due to change in F_I_O_2_ just before the blood sample was drawn, lack of SpO_2_ data, and a history of management with veno-arterial extracorporeal membrane oxygenation. In addition, patients with a difference of > 10% in SpO_2_ and SaO_2_ values were excluded due to the possibility of outliers. For validation purposes, we added the analysis of the raw data, including data of the possible outliers.

Patient baseline characteristics, including age, sex, height, body weight, body mass index, disease classification, and the use of veno-venous extracorporeal membrane oxygenation, were collected from electronic medical records. In addition, the results of blood gas analyses, including pH, PaO_2_, arterial carbon dioxide tension, haemoglobin, and SaO_2_ values, were recorded. Furthermore, the PaO_2_/F_I_O_2_ ratio and severity of hypoxaemia were determined. Underlying medical histories were obtained, including information on hypertension, ischaemic heart disease, chronic heart failure, chronic obstructive pulmonary disease, cerebrovascular accidents, diabetes mellitus, and chronic kidney disease requiring haemodialysis. The Acute Physiology and Chronic Health Evaluation II^19^ was used to assess organ dysfunction. ICU stays and mechanical ventilation durations were evaluated. In addition, ICU and hospital mortality rates were assessed.

### Pulse oximeter and blood gas analysis measurements

SpO_2_ was simultaneously measured by the TL-271T3 (Nihon Kohden, Tokyo, Japan) attached to Hamilton G5 ventilator, and by the RD SET™ NEO (Masimo, Irvine, CA, USA). Seal-type sensor probes were used and attached on the same side of the patient’s hand. We replaced the sensors at least three times a day, and the sensors were replaced at the same time during study periods. The selection of fingers to use in sensor replacement was dependent on the bedside nurse’s decision. The number of blood drawings or blood gases analyses were recorded and the SaO_2_ and SpO_2_ were compared. The timing of blood gas analyses was at the physician’s discretion. Blood samples were immediately transferred and analysed by the RAPIDLAB1265 (Siemens Healthcare Diagnostics Inc., Tarrytown, NY, USA) or the ABL800 FLEX device (Radiometer Medical ApS, Denmark).

### Statistical analysis

The relationship between measured SaO_2_ and SpO_2_ was evaluated using a Bland–Altman plot. Bias was calculated as the difference between SpO_2_ and the actual SaO_2_ from each pulse oximeter. Precision was determined from the standard deviation of the calculated bias. The bias and precision of the differences between SpO_2_ and SaO_2_, upper and lower limits of agreement, and mean values of SpO_2_ were calculated using bootstrapping methods. Resampling was randomly done with replacement up to 10,000 repetitions.

The ability to detect SaO_2_ ≥ 98% was assessed using an AUC because SpO_2_ 97% is default upper range of SpO_2_ on INTELLiVENT-ASV^20^. The ability to detect SaO_2_ < 90% was also assessed using an AUC to detect hypoxemia.

To evaluate the differences in oxygenation, we established three categories of SaO_2_ ranges (SaO_2_ < 94%, 94% ≤ SaO_2_ < 98%, and SaO_2_ ≥ 98%). Among these groups, differences between SpO_2_ and SaO_2_ were evaluated using the student’s *t*-test. All analyses were performed using JMP 15 pro (SAS Institute Inc., Cary, NC, USA). Data are presented as means ± standard deviations (SD) or as percentages where appropriate. *P*-values < 0.05 were considered statistically significant.

### Prior presentations

We presented prelimited data of this article at the European Society of Intensive Care Medicine 31st Annual Congress in Paris, France, 17 August 2018.

## Supplementary Information

Supplementary Information 1.

Supplementary Information 2.
