# Supplementary material for: Accuracy of two pulse-oximetry measurements for INTELLiVENT-ASV in mechanically ventilated patients: a prospective observational study
Source: Sci Rep. 2021 Apr 26;11:9001. doi: 10.1038/s41598-021-88608-7 (PMC8076307; doi:10.1038/s41598-021-88608-7)
Supplement: Supplementary file 1 — Supplementary Information 1. [file 41598_2021_88608_MOESM1_ESM.doc]

**Supplementary Information**

**Accuracy of two pulse-oximetry measurements for INTELLiVENT-ASV in mechanically ventilated patients: a prospective observational study.**

Shinshu Katayama M.D., Ph.D.; Jun Shima M.D.; Ken Tonai M.D.; Kansuke Koyama M.D., Ph.D.; Shin Nunomiya M.D., Ph.D.

Supplementary Figure S1. Bland–Altman plot comparing SaO_2_ and SpO_2_ analysed from the raw data.

The horizontal axis represents (SpO_2_ + SaO_2_)/2. The vertical axis represents SpO_2_ – SaO_2_. The middle horizontal red line represents bias. The upper horizontal red dotted line represents the upper limits of agreement, and the lower horizontal red dotted line represents the lower limits of agreement. Black dots represent each test result. The left figure portrays Nihon Kohden SpO_2_, and the right figure portrays Masimo SpO_2_.

Supplementary Table S1. Bias and precision for each pulse oximeter analysed from the raw data (n = 10 000)

| **Blood Gas Analysis** | Mean (95% CI) |
| --- | --- |
| SaO_2_ | 95.70% (95.55–95.85) |
| **Nihon Kohden SpO_2_** |  |
| SpO_2_ | 96.4% (96.3–96.6) |
| Bias (SpO_2_ - SaO_2_) | 0.72% (0.61–0.82) |
| Precision | 2.07 (1.88–2.35) |
| Upper limits of agreement | 4.78 (4.29–5.43) |
| Lower limits of agreement | -3.34 (-3.79–-3.07) |
| **Masimo SpO_2_** |  |
| SpO_2_ | 96.8% (96.6–97.0) |
| Bias (SpO_2_ - SaO_2_) | 1.08% (0.96–1.20) |
| Precision | 2.42 (1.99–2.94) |
| Upper limits of agreement | 5.82 (4.86–6.96) |
| Lower limits of agreement | -3.66 (-4.80–-2.70) |

CI, confidence interval; SaO_2_, arterial oxygen saturation; SpO_2_, pulse-oximetric oxygen saturation.
