# Supplementary material for: Accuracy of two pulse-oximetry measurements for INTELLiVENT-ASV in mechanically ventilated patients: a prospective observational study
Source: Sci Rep. 2021 Apr 26;11:9001. doi: 10.1038/s41598-021-88608-7 (PMC8076307; doi:10.1038/s41598-021-88608-7)
Supplement: Supplementary file 2 — Supplementary Information 2. [file 41598_2021_88608_MOESM2_ESM.pptx]

## Slide 1
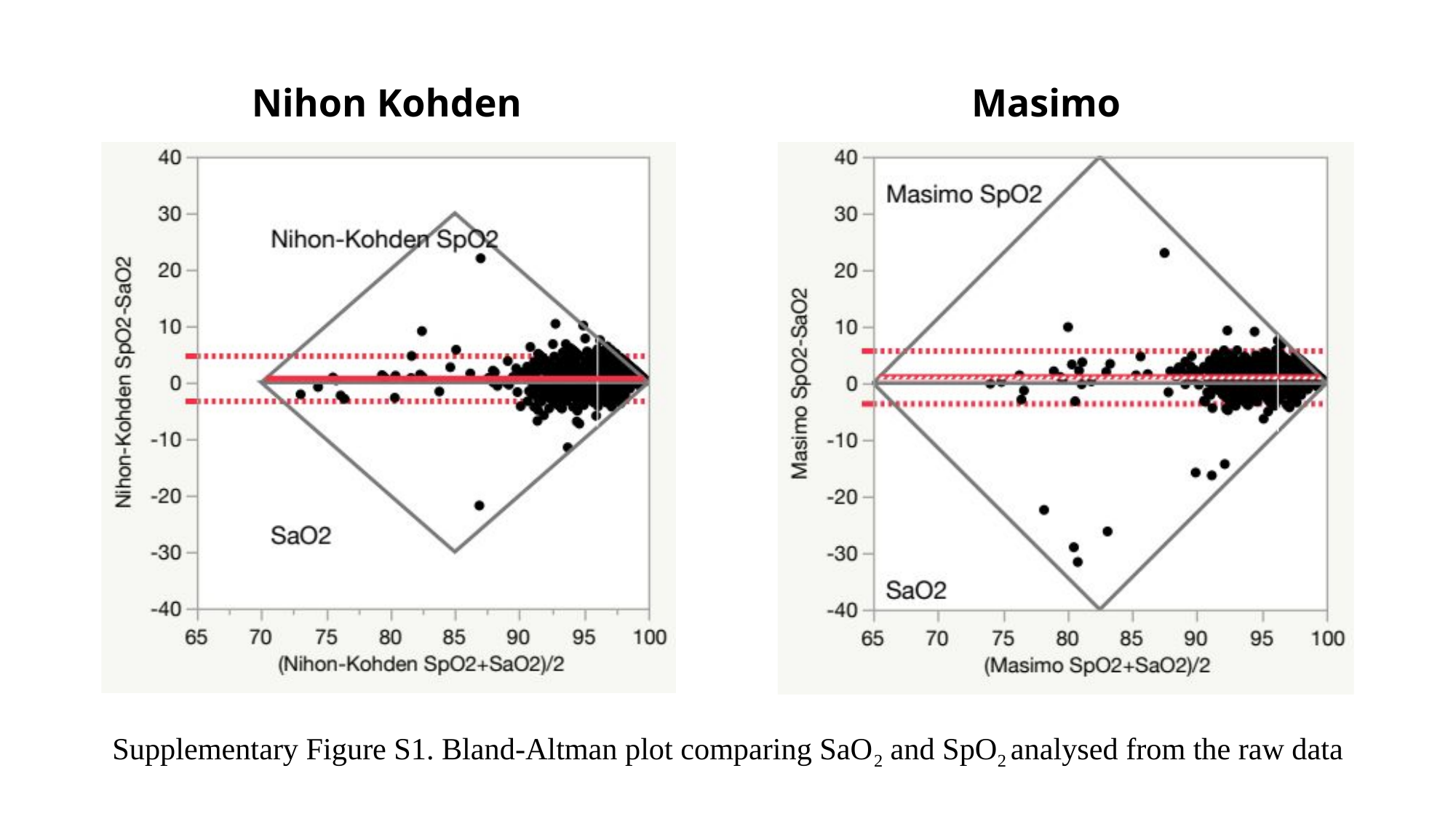

Nihon Kohden
Masimo
# Supplementary Figure S1. Bland-Altman plot comparing SaO2 and SpO2 analysed from the raw data
